# Hygiene in France
*Report of the Laws and Ordonnances in force in France for the Regulation of Noxious Trades and Occupations. By Waller Lewis, M.D.


**Published:** 1856-04

**Authors:** B. Daniel


### HYGIENE IN FRANCE.*

The disasters of our army before Sebastopol have taught us that in war it is not enough that men should be taught to fight, but that they must be fed and clothed also. It has also been gravely hinted that, in some particular preparations for a campaign, we might gain by the example of our Gallic allies; so that in future it may not happen that, through want of precaution, our generals should have to borrow French great-coats to cover English soldiers, or French mules to carry English forage. It is not, however, in war alone that our continental neighbours are able to afford us useful hints. In France, the art and science of hygiene has been prosecuted much further than in any other country; and the knowledge thus acquired has been freely applied by the French government in the construction of laws and regulations, having for their aim the improvement of the public health.

With the object of inquiring into the laws and ordonnances in force in France, in relation to trading occupations, Dr. Waller Lewis received instructions from Lord Palmerston to make a concise report on them; and, to obtain a more correct and practical acquaintance with their operation, he was authorised to proceed to France, where, through the kind offices of M. Billault, the Minister of the Interior, and of M. Collet Meygret, the Director-General of Public Health, he was enabled to inspect all the public and private industrial establishments that the time at his disposal admitted of. The result of these investigations is contained in a report which has been presented to both Houses of Parliament, and which we have now in review.

The following is a brief chronological record of French sanitary legislation. In 1486, potteries were suppressed in Paris by royal proclamation, on the complaint of the inhabitants of the neighbourhood. In 1567, chiffoniers, knackers, and tanners, were driven from the interiors of towns. Up to the 12th February, 1806, such establishments were not placed under the surveillance of the government; but at this date the prefect of police was empowered to visit, with or without previous notice, any workshop, manufactory, or laboratory, where occupations were carried on prejudicial

---

* Report of the Laws and Ordonnances in force in France for the Regulation of Noxious Trades and Occupations. By Waller Lewis, M.D.

to health, or likely to occasion a fire. Where it was intended to carry on such occupations, the sanction of the police was to be previously obtained. These regulations, however, were not efficiently carried out; and it was not until the decree of 15th October, 1810, and the ordonnance of the 14th January, 1815, that effectual measures were taken for the regulations of manufactures in the interest of public health.

These last two measures, which were drawn up by the Minister of the Interior, in consultation with the Academy of Sciences, are those which at present regulate these matters. Factories are therein divided into three classes: 1. Those which must be kept at a distance from private habitations, and require a certain amount of isolation; 2. Those which do not absolutely require being kept at a distance from private dwellings, but which, from the nature of the operations therein conducted, require an assurance that such shall not constitute a nuisance or a danger to the neighbourhood; 3. Works such as may remain without inconvenience near dwellings, but which should be subject to the surveillance of the police. It is the duty of the authorities to see that the provisions of the law are properly carried out. Before permission can be obtained to pursue any of the occupations in the first class, it is necessary to forward to the police two plans, one showing the connexion of the proposed works with the neighbouring houses and lands, and another showing its proposed internal arrangements. Public notices have to be placarded within a radius of three miles, stating the intention of applying for a license. The mayor of the commune in which it is proposed to erect the works, holds a meeting of the inhabitants, to ascertain their feelings on the subject, and sends the result of his inquiry also to the police. The prefect takes the documents to the Council of Health (and afterwards to the Council of Prefecture, if there is any opposition). These formalities being gone through, the prefect sends the whole of the documents to the Minister of Commerce, with his recommendations, whatever they may be; they are then submitted for the advice of the Council of State, and the minister proposes to the chief of the Government an order of refusal or authorisation, which the prefect is charged with executing. Authorisation of the second and third classes is obtained from the prefects and sub-prefects of the department or arrondissement, who consult with the Councils of Health.

The following are examples of classification. First class: public slaughter-houses (*abattoirs*), knackers' establishments, gut spinning, many chemical and metallurgical processes in which noxious gases are evolved, or which are liable to cause fires, such as the manufacture of fusees, chemical matches, or detonating powders; large furnaces giving off thick smoke, etc. etc. The second class includes examples where, from improved processes or arrangements, injurious tendencies are reduced, such as the manufacture of nitric acid from the decomposition of saltpetre by sulphuric acid in Wolf's apparatus, where gases are consumed or condensed instead of being diffused in the atmosphere, depôts of fresh hides, calcination of bones, candle making, tanning, sugar refineries, gas works. The third class contains slaughter houses in communes containing fewer than 10,000 inhabitants, cow keepers in towns with more than 5,000 souls, soap boiling, and many occupations which chiefly annoy by bad odours, noise, smoke, or danger of fire.

Of the abattoirs, placed in the first class, there are five; namely, Montmartre, Grenelle, Du Roule, Menilmontant, Villejuif, covering respectively, 8¾, 7¾, 5¾, 10¼, and 5½ acres. That of Montmartre is used more than all the rest together. They are situated on the right and left banks of the river, within the barriers, but a mile and three-quarters from the centre of the city. They were projected in 1810, but not completed until 1818. They are under the direct superintendence of the police; and in the decree which constituted them, and by which they are regulated, are numerous clauses relative to the public health. All the animals intended for the butchers' trade of Paris can only be slaughtered in one of these five public abattoirs. In addition to the buildings used for the lodging of the persons employed, the abattoirs of Paris are composed of four parts, entirely distinct:—

1. That where the animals about to be slaughtered are kept.
2. The abattoir proper, with all its accessories.
3. The place where are prepared the entrails of the slaughtered animals.
4. Lastly, those places where they prepare the tallow and fat.

In these establishments, besides the killing of animals, numerous processes are conducted, as the cooking the stomachs of oxen, cows, and sheep, intended for the tripe trade, and the preparation of sheep's and calves' feet, heads, etc., which necessitate isolated workshops. The melting of tallow, as it is not permitted to be carried on as a trade in Paris, also takes place in the abattoirs; but there are inconveniences attached to this system, which make its propriety questionable, the greatest of which arises from the offensive smells. An improved mode of melting the tallow which is obtained from the fatty parts of the animals, has been in some cases adopted, and is expected to be enforced in all these establishments; it consists in placing the fat, mixed with a certain proportion of water, and a fiftieth part of the same amount of sulphuric acid, in an air-tight closed copper vessel, heated by steam. In the old method, the fat is melted in large coppers, holding eight cwts., and placed over an open fire. The tallow obtained by these processes varies in appearance, being by the acid preparation whiter and rather firmer, and serving for the purpose of making candles as well as the other; but the sulphuric acid it contains renders it unfit for leather making. One of the most important considerations in the construction of these slaughter houses is, that there should be an unlimited supply of water, and of the means for conveying it away; 20,000 gallons a day are required by one of the Paris abattoirs alone. There are stringent regulations for the preservation of as much cleanliness as the nature of such places will permit. The contractors for the liquid, refuse, blood, etc., must remove it every day at an appointed hour. However repulsive the nature of these occupations may be, it does not seem (from the nature of the information given in a letter of Dr. Waller Lewis to Sir George Grey) to interfere with the health of the workmen; on the contrary, he states—"They are particularly healthy, being free from bowel complaints, rarely or ever having attacks of cholera in seasons of epidemics. This is a fact which has constantly been given to me in evidence, both in France and England, that workmen engaged in manufactories connected with dead animal matter, are particularly free from disorders of this class."

Knackers' establishments are placed in the first class; and, as it is considered that at Montfaucon 400,000 horses and 1,500,000 dogs and cats have been killed, the necessity of an active superintendence is apparent. At Aubervilliers, near Paris, on an average, 6,000 or 7,000 horses are killed; they cost from seven to 10 francs each, and the carcasses are made to realise from £3 to £4 each. Thus the hair, skin, blood, muscular parts, entrails, tendons, fat, even the shoes and nails, are all utilised, some for food of animals, others for commerce or for agriculture. The skin and hair are first removed and disposed of; then the animals are cut up and boiled in air-tight iron cylinders, under a high temperature; the bones are afterwards separated from the flesh, and are sold to the manufacturers of animal black; the flesh is dried, ground, and sifted, and is converted into manure. The intestines of the animals, which have to be converted into receptacles for sausage-meat, and twisted into musical cords, have to undergo scraping, cleansing, inflating, deodorising, and bleaching, by exposure to the fumes of sulphur. The stench is said to be so horrible in the places where these operations are carried on, as to require some little courage on the part of any one desirous of visiting them. Nevertheless, Parent Duchatelet affirms that the emanations from these gut establishments may be breathed with as perfect impunity as the most agreeable odours. On the other hand, MM. Chevalier and Guérard report that, in the visits they have repeatedly made to these establishments, they have been informed that workmen, at the commencement of their work, are in a few days attacked with fever, and derangement of their digestive functions, which require a course of purgatives for their relief. The opinion of the latter gentlemen does not militate against common sense, which would naturally induce us to believe that that which is so repugnant to the smell, and revolting to the sight, may be injurious to the health. Chlorides, carbon, and the disinfecting powder of MM. Payen and Salmon (composed of calcined earth containing vegetable matter, and acting by means of the finely divided charcoal it contains), are used to diminish the smell arising in gut spinning establishments. The production of animal charcoal from bones is carried on in France so largely, as to require large importations of bones from abroad. The charcoal is chiefly used in the clarification of sugars and vinegars. "The clarification process for sugar consists in adding three kilogrammes of animal charcoal, in fine powder, to a hundred kilogrammes of raw sugar, with one or two kilogrammes of albuminous matter, coagulable by heat. Bullocks' blood, beaten, and thus deprived of its fibrin, then mixed with four times its volume of water, is made use of for this purpose."

Unlike London, Paris does not possess the advantage of a system of drainage, and all the night-soil has to be removed by manual labour; which is done either by a periodical emptying of the cesspools, or by means of what are termed *fosses mobiles*, or removable receptacles. In the forest of Bondy, near Paris, is a large establishment for the manufacture of artificial manure, whither the night-soil of nearly the whole of the capital is conveyed. This removal is contracted for by Messrs. Richer and Co.; and the town of Paris re ceives eighty-five *centimes* (about 8*d.*) for every cubic *mètre*

(220 gallons) of solid and liquid that enters the factory. At the present time, when so many projects are advanced for the interception of the sewage which flows into the Thames, it is worth while to bear in mind that, if an establishment of this kind can afford to pay for it, under the difficult circumstances under which it is transported, it is extremely probable that, if means could be adopted for the reception of the London sewage at the points where the sewers open into the river, and if from thence it could be conveyed away by barges, the utilisation of this substance would probably more than pay for the expense of its collection. The liquid portion of the sewage could be allowed, by a process of filtration, to run off by a subaqueous canal to any distance, leaving the solids in the reservoir for removal. In the French establishment alluded to, sulphate of ammonia is obtained from the liquids; the solids are dried, and used as a manure in powder. The smell of this factory extends to a great distance, and is very bad, and much complained of in the neighbourhood.

Cow-keeping has not evaded the vigilance of the French government. This is a very important subject, since the condition of the milk is much dependent upon the healthy state of the cows. The regulations which exist have for their object to prevent overcrowding of the animals, to ensure cleanliness and a sufficient ventilation in the stables.

The influence of chemical processes used in various manufactures, in affecting the health of the manufacturer, does not escape attention in the French capital; and the precautions enforced are in many cases attended with the best results.

At the manufactory of white lead in Paris, conducted by M. Besançon, all the men employed, according to Dr. Waller Lewis and M. Trebuchet, the Chef du Conseil de Salubrité, at the Prefecture of Police, appeared in perfect health; there was no blueness of the gums, nor were any of the signs of lead poisoning to be observed; nor were complaints ever heard from the workmen since the occupation had been carried on under the existing improvements. This is a great result, considering that 5,000 kilogrammes (about 98½ cwts.) of white lead are made and ground daily. The nature of the preparations are as follow; we quote Dr. Lewis's words:—

"No man is allowed, under any circumstances, to continue at this work more than seven days consecutively. In quitting their work, the men are obliged to wash their hands and face in water containing a solution of sulphuret of potassium; secondly, in water holding clay in suspension; and thirdly, in pure water. They must use these ablutions whenever they cease work, before taking a meal, or retiring for the day. These are among the regulations of the police, and are made known to the workmen by placards printed and fixed up in various parts of the factory.

"In the subsequent process, the lumps of white-lead are ground under water, and no powder, vapour or smell can thus escape. The white lead is afterwards separated from water by pressure, placed on plaster of Paris, and exposed to hot air until it is quickly made anhydrous; after which, it is placed into receiving boxes, where it is broken into small pieces, by a few gentle taps of a hammer, without creating a dust. After this, it is immediately mixed with oil, with which it is ground. No lead is sold in the factory unmixed with oil.

"After the drying, as the lead before admixture with the oil must be in the form of an impalpable powder, it is passed through very fine sieves. This process—which in many manufactories is still done by the workmen themselves, who shake the powder through the sieves—is performed at M. Besançon's factory in hermetically-sealed closets or small rooms, in sieves supplied with the lead, and having their shaking motions communicated to them by the steam-engine that works their grinding-mills."

In a sanitary point of view, the difference between the English and French modes of dressing leather deserves also a brief notice. In converting skins into leather, the first thing is to remove the hair, which is done in both countries by the action of lime; in the next process, by which the lime is removed from the skins, the method differs. In England it is done by the action of the putrified dung of animals, particularly of dogs; and it is said that three or four hundred men are occupied in the streets of London in picking up this excrement and collecting it in a bag, which they carry at their side. Their collection is purchased by the tanners under the name of "pure". It is kept in mass until the emanation of carbonic acid, sulphuretted and phosphuretted hydrogen, indicate its putrefaction and fitness for use. The smell necessarily becomes a disgusting nuisance, and pervades the whole establishment. In France, the process of getting rid of the lime in tanning is simply effected by complete and free washing in liberal quantities of water, by pressure, and by use of an engine for the purpose, known by the name of "appareil purgeur, *Systeme de Artus frères, vendeurs de l'Appareil,* 1853." By the aid of two such machines, 1,200 smaller skins can be finished off in a day.

*B. Daniel.*

